# Application of Probiotic Yeasts on *Candida* Species Associated Infection

**DOI:** 10.3390/jof6040189

**Published:** 2020-09-25

**Authors:** Lohith Kunyeit, Anu-Appaiah K A, Reeta P. Rao

**Affiliations:** 1 Department of Microbiology and Fermentation Technology, CSIR- Central Food Technological Research Institute (CFTRI), Mysuru 570020, India; lohichanthala@yahoo.in (L.K.); anuappaiah@cftri.res.in (A.K.A.); 2 Academy of Scientific and Innovative Research (AcSIR), Ghaziabad 201002, India; 3 Department of Biology and Biotechnology, Worcester Polytechnic Institute, Worcester, MA 01609, USA

**Keywords:** *Candida albicans*, non-albicans *Candida* species, *Candida auris*, *Saccharomyces boulardii*, *Saccharomyces cerevisiae*, aromatic alcohols

## Abstract

Superficial and life-threatening invasive *Candida* infections are a major clinical challenge in hospitalized and immuno-compromised patients. Emerging drug-resistance among *Candida* species is exacerbated by the limited availability of antifungals and their associated side-effects. In the current review, we discuss the application of probiotic yeasts as a potential alternative/ combination therapy against *Candida* infections. Preclinical studies have identified several probiotic yeasts that effectively inhibit virulence of *Candida* species, including *Candida albicans*, *Candida tropicalis*, *Candida glabrata*, *Candida parapsilosis*, *Candida krusei* and *Candida auris.* However, *Saccharomyces cerevisiae* var. *boulardii* is the only probiotic yeast commercially available. In addition, clinical studies have further confirmed the in vitro and in vivo activity of the probiotic yeasts against *Candida* species. Probiotics use a variety of protective mechanisms, including posing a physical barrier, the ability to aggregate pathogens and render them avirulent. Secreted metabolites such as short-chain fatty acids effectively inhibit the adhesion and morphological transition of *Candida* species. Overall, the probiotic yeasts could be a promising effective alternative or combination therapy for *Candida* infections. Additional studies would bolster the application of probiotic yeasts.

## 1. Introduction

The fermented foods are a rich source of beneficial microorganisms, and they have a long history of exhibiting health benefits, particularly *S. cerevisiae* and lactic acid bacteria (LAB). Their safety is evidenced by consumption of fermented foods and beverages over centuries. Today, it is well accepted that the rich microbial profile of fermented food provides more than just nutrition. For example, functional activity of microorganisms in food helps enhance the bio-availability of micronutrients, improving the sensory quality and shelf life of the food, degrading anti-nutritive factors (such as trypsin inhibitors and phytate degradation), enriching antioxidant and antimicrobial compounds, and fortifying health-promoting bioactive compounds [1,2]. These attractive microbial activities in the fermented foods have been a draw in the field of probiotics.

Characteristics of bacterial strains such as *Lactobacillus* and *Bifidobacterium* species have been extensively studied and commercially available as probiotic supplements. Yeasts, which are also common in fermented foods, remain largely unexplored for probiotic potential. We and other researchers have observed that yeasts that originate from fermented sources such as apple cider, wine, fermented coconut palm, and fermented dairy products survive the harsh condition of the gastrointestinal (GI) tract and retain the ability to attach to intestinal epithelium [3,4,5]. More recently, live bacteria have been used in fecal transplants to prevent and/or treat several GI complications [6]. The probiotic bacteria, such as lactic acid bacteria (LAB) and *Bifidobacterium* species, have effectively treated several GI complications, including candidiasis [7,8]. However, other than *Saccharomyces boulardii,* potential probiotic yeasts such as *S. cerevisiae* and several other non-*Saccharomyces* yeasts are largely unexplored use as biotherapeutics, specifically for *Candida* infections. In reviewing the current literature here, we focus on the biotherapeutic potential and mechanism(s) of action of beneficial yeasts against *Candida* infections.

The vast majority of fungal infections are caused by *Candida albicans*, a polymorphic commensal yeast as well as some non-albicans *Candida* species. Disease range from superficial infections, such as cutaneous and mucosal, to life-threatening bloodstream infections (BSI), or invasive deep tissue infections. Superficial infections usually affect the nails, skin, and mucosal membrane of the host and are recalcitrant to treatment. For example, vulvovaginal candidiasis (VVC) has infected 75% of women population at least once in their lifetime. Furthermore, a small population (5–8%) suffers from at least four recurrent VVC per year [9].

Bloodstream infection (BSI) and other invasive *Candida* infections cause high morbidity and mortality especially among immune-compromised patients [10]. *Candida* species are the fourth-leading cause of nosocomial infections in the world, and *Candida* BSI attributes to 35% mortality rate in all the *Candida* associated infections [11]. Furthermore, the National Nosocomial Infection Surveillance System (NNIS), USA, has revealed total 27,200 nosocomial infections between January 1980 through April 1990, among these *C. albicans* and non-albicans *Candida* species were involved total 19,621 (72%) of the overall infections [12].

Though *C. albicans* is a major commensal yeast flora of the GI tract, non-albicans *Candida* species such as *Candida glabrata*, *Candida tropicalis*, *Candida parapsilosis,* and *Candida krusei* have been frequently identified in a healthy individual’s gut. On the other hand, among 15–20 pathogenic non-albicans *Candida* species, *Candida glabrata*, *Candida tropicalis*, *Candida parapsilosis,* and *Candida krusei* are predominant constituting 35–65% of the overall infections [13]. As an opportunistic pathogen, certain groups of immune-compromised individuals have a higher susceptibility towards *Candida* infection. Invasive *Candida* infections are also closely associated with advanced medical techniques such as medical implants and stents [14]. For instance, the patients who are on antibiotic therapy and chemotherapy, central venous catheters, total parenteral nutrition, extensive surgery, burns, renal failure and hemodialysis, or mechanical ventilation are at a major risk for superficial and invasive *Candida* infections [14].

## 2. Morphological Transition and Metabolic Flexibility Promote Virulence of *Candida* In Vivo

As a polymorphic yeast, *C. albicans* and few non-albicans *Candida* strains, such as *C. tropicalis* and *C. glabrata,* exhibit multiple morphological structures such as yeast form, germ tubes, pseudo-hyphae, and/or hyphae that play a key role in the infection. For example, filamentous morphology is well-known for epithelial invasion and is primarily involved in biofilm formation [15]. Yeast form cells are planktonic and are important for dissemination. Once they attach, they initiate germ tubes, pseudo-hyphae, and/or hyphae that enhance adhesion to surfaces. Attachment to abiotic surfaces initiates biofilm formation. Biofilms on implanted medical devises may lead to invasive fungal infections—a major risk factor for *Candida* infection-associated mortality [16]. Attachment to live cells (such as epithelium) causes damage, evokes an immune response and ultimately gains access to deeper tissues. Therefore, the polymorphism of *Candida* is an important consideration in its infectious outcomes.

The host’s innate immunity is a major factor in fungal clearance, normally through a process called phagocytosis where immune cells ingest and biochemically eliminate the pathogens [17]. However, the switch from yeast to filamentous form is a common escape mechanism of *Candida* species [18]. Therefore, *C. albicans* filament has less susceptibility for phagocytosis by innate immune cells than the yeast form [19]. In addition, metabolic flexibility of *C. albicans* facilitates colonization by adapting to varying nutritional availability [20]. For instance, in case of *Candida* meningoencephalitis (*Candida* infection in brain tissue), glucose and vitamins are the major nutrient sources for the pathogen, while in liver, it utilizes glycogen as a nutrient source [9]. A study revealed that adaptation to alternative carbon sources such as lactate and other nutrient sources increased environment stress response and virulence [21]. All of these attributes make *C. albicans* and non-albicans *Candida* species a unique pathogen among the microbial community.

## 3. Drug Resistance Is a Major Hurdle to Antifungal Therapy

Antifungal drugs used to treat *Candida* associated infections, work either by killing or inhibiting the growth of *Candida* species. A sparse number of antifungal classes such as polyenes, azoles, and echinocandins are used depending on conditions of invasive *Candida* infections [22]. Multiple *Candida* strains have already developed resistance to these drugs making this a public health threat [23]. For example, surveillance data from health-care facilities revealed widespread fluconazole resistance among clinical isolates of both *C. albicans* and non-albicans *Candida* strains [24,25,26]. Azoles such as fluconazole is a first-line antifungal drug that is used extensively for therapy and prophylaxis against *Candida* infections. Several resistant mechanisms have been connected to drug-resistant *Candida* species including overexpression of drug efflux pumps, alteration in drug targets, and changes in membrane sterol composition [22]. The structural heterogeneity of *Candida* biofilm has a major significance in clinical context due to higher resistance against most antifungal agents. Furthermore, these drugs can be toxic for the patients with several side effects that include GI disturbances, hepatotoxicity, and neurotoxicity due to their target resemblance to its host cell, antifungal metabolism in liver and cross drug interaction in the host [27,28].

More recently, multi-drug resistant *Candida auris* has emerged as a “super bug” posing significant clinical challenges and a major threat to public health. *Candida auris,* is often involved in the nosocomial bloodstream infection world-wide [29]. *C. auris* has been shown to last in the hospital settings and spread from person-to-person by direct contact or contaminated surfaces [23]. In addition, *C. auris* is closely related with *Candida haemulonii* and is often misidentified as such. This requires a specialized laboratory method for identification [23], further delaying implementation of infection control. Therefore, now more than ever, there is an urgent need for effective alternatives to conventional modes to treat *Candida* infections.

Some attempts have been made to using specific diets that avoid high sugar-containing food such as bakery products, milk, and dairy product. The claim is that it reduces *Candida* colonization of the GI tract [30]. Intestinal overgrowth of *C. albicans* contributes to Crohn’s disease that affects 1.6 million Americans [31,32]. *C. albicans* overgrowth is caused by an imbalance in the intestinal microbiota and host immune status. To restore the balance and modulate host immunity, foods rich in antioxidants and other nutritional supplements have been suggested [30,33]. More recently, studies on the human microbiome have opened new insight into the role of the resident gut microbiota in physical health and mental wellbeing. Applications of beneficial microbes as fecal transplants [34] or fermented milk products [35] for the treatment of irritable bowel syndrome (IBS) and irritable bowel disease (IBD) has gained traction. Here we discuss the potential of probiotic yeasts against *Candida* virulence and pathogenesis.

## 4. Use of Probiotics as Biotherapeutics

As stated by Hippocrates, “let food be thy medicine and medicine be thy food”. Today, the idea of food and/or diet is not just extended towards mere survival or hunger satisfaction. The health-conscious population deeply cares about additional aspects including health improvement and prevention of diseases. In this context, functional food plays a significant role where, the concept of food has not only intended to provide humans with necessary nutrients, but also to prevent diseases and increase physical and mental well-being. Probiotic, considered as a functional food, is mostly consumed in the form of traditional fermented food products such as milk products, fermented vegetables, and meats [36].

Probiotics are defined as “live microorganisms which, when consumed in adequate amounts, confer health benefits on the host” [37]. The archived scientific documents have explained the diverse positive effects of probiotics on a wide range of diseases and disorders including lactose indigestion, diarrhea, immune modulation, inflammatory bowel syndrome, constipation, infection, allergy, serum cholesterol, blood pressure, and reduction of urinary tract infections [38]. In addition, the Human Microbiome Project by National Institute of Health (NIH), USA, changed the views on beneficial microbial research; it exposed the influence of gut microbiome and human health during various infections and disease conditions including, mental health.

## 5. Interaction of Probiotics Yeast and *Candida* Species

Several reports suggest that probiotic bacteria are effective against GI complications such as diarrhea, leaky gut syndrome, as well as *Helicobacter pylori* and *Clostridium difficile* infections [39,40]. However, *Saccharomyces cerevisiae* var. *boulardii* is the only yeast currently available for human use as probiotics. Its efficacy against *Candida* has been explored previously. Specifically, pathogen-free mice that were infected with *C. albicans* and subsequently treated with *S. boulardii* prevented the translocation of *Candida* to internal organs [41,42,43]. These groups further confirmed that *S. boulardii* effectively reduced *C. albicans* translocation colonization and inflammation in in vivo models.

Clinical reports around the use of probiotic yeasts are limited. One study, reports that oral administration of *S. boulardii* to infants reduced the fungal colonization and invasive fungal infections [44]. Another study conducted in preteen children focused on the effects of probiotics against *Candida* infection. They used a probiotic cocktail of yeast and bacteria in combination with prebiotics and demonstrated a reduction in colonization of *C. albicans* [45].

## 6. Probiotic Yeasts Exhibit Multiple Inhibitory Mechanisms against *Candida* Species

Pre-clinical and/or clinical studies indicate that *S. boulardii* and other potential probiotic yeasts ameliorate complications associated with *Candida* infection by mechanisms outlined in Table 1. However, there was a lack of specific mechanistic insights on how these probiotic yeasts interact with *Candida* species especially in the context of a live host. Pathogens in GI tract induce necrosis and apoptosis of intestinal epithelia by reducing the production of mucin or its degradation. Pathogens also downregulate IgA and other proteins of the tight junction thereby increasing intestinal permeability [46,47]. *S. boulardii* has been shown to increase IgA production in *Clostridium difficile* colitis and antibiotic-associated diarrhea in mice model [48]. *S. boulardii* also decreases epithelial necrosis, apoptosis, and increases the production of antioxidant enzymes such as superoxide dismutase, catalase, and glutathione peroxidase in mouse models in necrotizing enterocolitis in mice [49]. In addition, *S. boulardii* activates the intestinal epithelial restoration in GI tract [50]. Together these cellular responses may contribute to its beneficial properties and prevent *Candida* infection.

### 6.1. Immunogenic Response and Anti-Virulence Ability of Probiotic Yeasts

Since resistance to antifungal drugs has emerged as a significant problem, researchers have explored alternative means of treating recalcitrant fungal infections. Modulation of host immunity is one avenue that is being considered as an alternative [56,57]. For example, *S. boulardii* has been shown to reduce pro-inflammatory cytokines such as IL-1β and TNF, and increase anti-inflammatory cytokines IL-4 and IL-10 during *Candida* infection [42,58]. Other alternative therapies target virulence strategies such as adhesion and filamentation of *C. albicans* [59]. These maybe used to treat abiotic surfaces to deter microbes from binding. Probiotics also have the ability to inhibit virulence factors of the pathogen. We and others have demonstrated that cells, as well as the cell-free secretome of probiotic yeasts such as *S. boulardii, S. cerevisiae,* and a non*-Saccharomyces* yeast *Issatchenkia occidentalis* inhibit adhesion, filamentation, and biofilm development of *C. albicans* [52] and other non-albicans *Candida* species such as *C. tropicalis, C. krusei, C. glabrata,* and *Candida parapsilopsis.* Biofilms are complex multispecies structures that include *C. albicans* among other microbes [60,61]. Probiotics yeasts have been shown to be effective against fungal biofilms composed of *C. albicans* and non-albicans *Candida* species [53]; however, no studies have been focused on their efficacy on cross-kingdom biofilms. These studies implicated the involvement of yeast metabolite(s) in inhibiting adhesion and/ or morphological transition in vitro [53]. These studies also indicate that probiotic yeast affect a broad spectrum and not limited to *C. albicans;* rather, it can inhibit virulence across the *Candida* genus.

Cultured intestinal epithelial models such as Caco-2, Intestin 407 and HT-29 have been extensively used to study microbial interactions or host-microbe interactions. These cell lines recapitulate various features of the intestinal epithelial surface including the formation of villi, production of mucus, and antibodies such as IgA [62]. We and others have demonstrated that probiotic yeasts effectively reduce adhesion of *C. albicans* and non-albicans *Candida* species to these cultured epithelial cell lines [52,53]. In addition, yeast *S. boulardii* has been shown to pose a barrier and preserve the integrity of the epithelium by the reduction of pro-inflammatory cytokines in the intestine [40,52].

**Figure 1:**
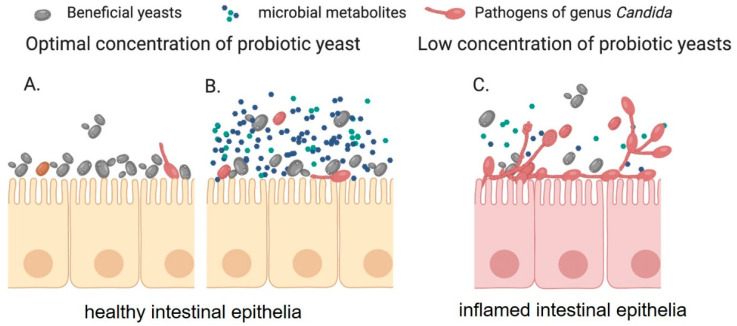
Probiotic yeast either form a physical barrier on epithelial surfaces (**A**) or secretes bioactive metabolite (**B**) to inhibit the adhesion and morphological transition of *Candida* species on epithelial cells. Further, suitable probiotic yeasts cell number is required for the effective inhibition of *Candida* virulence in the host GI tract (**C**).

Even though live probiotic cells are known to play a significant role in preventing virulence of *C. albicans*, the role of exact cellular components involved are less investigated. For example, administration of cell wall components of *S. cerevisiae* reduced the *Candida* associated inflammation and colonization in animal models [55]. Interestingly, one of the four *S. cerevisiae* strains used in this study (strain Sc-4) increased the mortality and inflammation in the host, suggesting strain-specific effects of the probiotic yeasts against *Candida* species [55]. Such strain specificity as also been reported in the interaction of *Lactobacillus* strains with *C. albicans* [63]. Furthermore, heat-killed *S. cerevisiae* reduced the vaginal colonization of *C. albicans* when applied against vaginal candidiasis in a murine model [54]. These effects could either mediated by yeast cell wall components such as β-glucan or simply that the biomass of heat-inactivated probiotic cells form a physical barrier that occludes host factors that facilitate *C. albicans* attachment (Figure 1A) [54].

### 6.2. Role of Small Bioactive Metabolites in Probiotic Action

Beneficial microbes or probiotics in the intestine are thought to control pathogen overgrowth by competing for limited nutrients. There is a growing body of literature that supports the notion that inhibitory function is primarily mediated by secreted small molecules with suitable probiotic cell number (Figure 1B,C) [53,64]. Microorganisms produce metabolites that have been shown to alter the course of an infection by synergistic or antagonistic interactions with infectious agents. Such metabolites include hydrogen peroxide, bacteriocins, and organic acids that effectively inhibit the virulence and growth of various *Candida* species [64,65] (Table 2). On the other hand, few interesting microbial metabolites, such as tyrosol and indole-3 acetic acid, trigger the filamentation in *C. albicans* [66,67]. Small molecules derived from bacteria have been evaluated for activity against *Candida* virulence and pathogenesis. For example, lectins of lactobacilli and bifidobacterial strains isolated from humans have been shown to inhibit the growth of drug-resistant *C. albicans* [68]. The Gram-positive pathogenic bacteria, *Enterococcus faecalis,* produces a peptide called EntV which has been shown to reduce *C. albicans* virulence [69]. Furthermore, organic acids such as acetic acid and lactic acid have been shown to enhance antifungal treatment of *C. albicans* and *C. glabrata* [70]. Many *Lactobacillus*, *Bifidobacterium,* and yeasts strains produce these organic acids. *S. boulardii* produces several bioactive compounds such as *Saccharomyces* anti-inflammatory factor (SAIF), anti-toxin factors, short-chain fatty acids, bioactive proteins of 54 kDa, and 120 kDa which play a major role in preventing bacterial infections [38,71]. However, there has been very limited knowledge on probiotic yeast metabolites on *Candida* species. Recently a group showed that yeast *S. boulardii* metabolite capric acid (Decanoic acid)—a saturated fatty acid, inhibits the filamentation of *C. albicans* interaction [52]. 

In natural habitats, potential interaction of microbial communities has been a key element for the ecological dynamics. Bacteria and eukaryotic microorganisms exhibit both symbiotic and/or antagonistic interaction in the natural environment. In fact, *C. albicans* co-exists with other non-albicans *Candida* species or bacteria in the biofilm as well as the human GI tract. These inter-species interactions between C. albicans and other microbes typically affect filamentation of *C. albicans*. For instance, certain secretory molecules of *Salmonella typhimurium* and *Streptococcus mutants* inhibit cell growth and filamentation of *C. albicans* in the co-culture conditions [72,73]. Another well studied bacterium is *Pseudomonas aeruginosa*, where bacterial toxin phenazine inhibits the filamentation of *C. albicans* [74,75]. 

The morphological transition of yeast has been controlled by cell density and/or quorum sensing molecules. Apart from bacteria, the quorum sensing mechanism is also well studied in yeast such as *C. albicans* and *S. cerevisiae*. Farnesol and tyrosol are known cell density molecules in *C. albicans* which controls the morphological transition. Similarly, yeasts such as *S. cerevisiae* and other many non-*Saccharomyces* yeast produce alcoholic signaling molecules called phenylethanol and tryptophol. An abundant usage and availability of well-curated genetic database indicate that *S. cerevisiae* has gained more attention on quorum sensing mechanisms than the non-*Saccharomyces* yeast strains. There are few studies claiming that factors such as low nitrogen content and cell density play a significant role in the production of phenylethanol and tryptophol in *S. cerevisiae* and regulates its morphological transition mechanism [76]. Furthermore, these signal molecules are controlled by the expression of *ARO8*, *ARO9,* and *ARO10*, where *ARO8* and *ARO9* encode the aromatic aminotransferases and *ARO10* encodes the aromatic decarboxylase reaction [77,78].

Several research groups have predicted and/or observed an antagonistic nature of aromatic alcohols, phenylethanol, and tryptophol against fungi. Winters et al., (2019) reported that high concentrations of *S. cerevisiae* inhibited non-*Saccharomyces* strains in mixed cultures and under fermentation conditions [78]. Although there were direct evidence of inhibition due to these secondary metabolites, commercially procured phenylethanol and tryptophol have been shown to inhibit filamentation of *C. albicans* [77]. This result is bolstered by the observation that administration of tryptophol enhances survival of *Galleria mellonella* larval that are infected with *Candida* [80]. Furthermore, a cocktail of phenylethanol, isoamyl alcohol, E-nerolidol, and farnesol provides protection against *Candida* infection in a murine model of infection [81]. Together these studies establish a paradigm for inhibition of fungal virulence that is mediated by aromatic alcohols.

## 7. Gaps in our Understanding of Biotherapeutic Application of Probiotics for *Candida* Infection

Probiotic yeasts yield several positive outcomes in in vitro, ex vivo, and in vivo readouts during colonization of *Candida* species. Information about their effect during systemic infection is an area that needs further investigation. Numerous animal and handful of clinical experiments have revealed that probiotics and metabolites such as short-chain fatty acids, tryptophol and phenylethanol play an abundant role in human health and diseases. However, the origin of these metabolites is ill-defined and their effects on clinical manifestations of *Candida* infection need further investigation. These studies would provide substantive information to improve biotherapeutic properties of beneficial microbes against *Candida* infections.

Emergence of drug resistance and complications associated with side effects have sparked interest in alternative therapies. Applications of food-derived yeasts have been shown to have positive outcomes against *C. albicans* and non-albicans *Candida* species virulence and infection in pre-clinical and clinical settings. Food-derived beneficial yeasts are also generally safe and pose an effective alternative to traditional antifungals. They may also be used in combination therapy with conventional antifungal drugs since the synergistic effect of probiotics and antifungal agents would prevent emergence of drug resistance. 

## Figures and Tables

**Table 1:** List of probiotic yeasts and its mechanisms against virulence and pathogenesis of *Candida* species.

Probiotic Yeast Strains	Mechanisms of Probiotic Yeasts against *Candida* Species Virulence and Pathogenesis
*S. boulardii*	Inhibits *C. albicans* and non-albicans *Candida* species include *C. tropicalis*, *C. krusei*, *C. parapsilosis*, *C. glabrata,* and *C. auris* adhesion, biofilm formation and/or filamentation, in in vitro, ex vivo, in vivo, and clinical settings [51,52] Secrets small bioactive molecules [52,53] Reduces inflammatory cytokines TNFα and INF γ in colon epithelial [41] Prevents the *C. albicans* translocation in GI tract [43]
S. cerevisiae *	Inhibits *C. albicans* and non-albicans *Candida* species adhesion, colonization, biofilm formation, and filamentation in in vitro, ex vivo, and in vivo models [53,54] Inhibits the *Candida* adhesion to epithelial cells by initiation of co-aggregation [54] *S. cerevisiae* form a barrier over the biotic surfaces and inhibits the *Candida* adhesion [54] Reduces virulence gene expressions of *C. albicans* during infection [54] Secrets bioactive molecules [53] Decreases the pro-inflammatory cytokine TNF-α and enhances IL-10 expressions in the host [55] Decreases the colorization and host cell damage during the infection [53,54] β-glucan decreased intestinal inflammation [55]
I. occidentalis *	Inhibits *C. albicans* and non-albicans *Candida* species such as *C. tropicalis*, *C. krusei*, *C. parapsilosis*, *C. glabrata,* and *C. auris* adhesion, colonization, biofilm formation and/ or filamentation in vitro, ex vivo, in vivo models [53] An unidentified metabolite (s) inhibits virulence of non-albicans *Candida* species [53]

* Potential probiotic yeast, not commercialized.

**Table 2:** Microbial metabolites and its functions against *Candida* species.

Microbial Strains	Bioactive Metabolite	Functions
*S. boulardii* [52]	Short-chain fatty acids (capric acid)	Filamentation inhibition, and antifungal activity against *C. albicans*
*S. cerevisiae* [53,54]	Unknown	Adhesion and filamentation inhibition
*I. occidentalis* [53]	Unknown	Adhesion and filamentation inhibition
*Lactobacillus acidophilus, L*. *crispatus, L*. *vaginalis* [65,68]	Lectins, hydrogen peroxide, lactic acid	Inhibit cell growth of *C. albicans*
*Bifidobacterium adolescentis, B. bifidum, B. gallinarum* [68]	Lectins	Inhibit cell growth of *C. albicans*
*Enterococcus faecalis* [69]	Peptide EntV	Filamentation inhibition
*Pseudomonas aeruginosa* [75,79]	Phenazine, 3-oxo-C12 homoserine lactone	Filamentation inhibition
*Salmonella typhimurium* [72]	Unknown	Inhibit cell growth and filamentation
*Streptococcus mutants* [73]	Unknown	Inhibit cell growth and filamentation
